# Association of the *GHRd3* polymorphism with adult height and type 2 diabetes in a Saudi Arabian population from Jazan Province: A case-control study

**DOI:** 10.12669/pjms.40.3.7686

**Published:** 2024

**Authors:** Reham G. Al-hakami, Alaa Y. Mashraqi, Rola Ali Atiah Shannaq, Yahia A. Kaabi

**Affiliations:** 1 Reham Ghazi Al-hakami, BSc. Department of Medical Laboratory Technology, College of Applied Medical Sciences, Medical Research Center, Jazan University, Jazan, Saudi Arabia; 2 Alaa Yahia Mashraqi, BSc. Department of Medical Laboratory Technology, College of Applied Medical Sciences, Medical Research Center, Jazan University, Jazan, Saudi Arabia; 3 Rola Ali Atiah, BSc. Department of Medical Laboratory Technology, College of Applied Medical Sciences, Medical Research Center, Jazan University, Jazan, Saudi Arabia; 4 Yahia Ali Kaabi, PhD. Department of Medical Laboratory Technology, College of Applied Medical Sciences, Medical Research Center, Jazan University, Jazan, Saudi Arabia

**Keywords:** Growth hormone receptor (GHR), GHRd3 polymorphisms, Adult height, Type-2 diabetes mellitus, Genotyping

## Abstract

**Objectives:**

This study investigated the association of the GHRd3 polymorphism with height and type-2 diabetes mellitus (T2DM) in Saudi Arabia.

**Methods:**

This case-control study included a total of 284 participants, divided into healthy controls (n = 142) and patients with T2DM (n = 142), recruited from Jazan University Hospital, southwest of Saudi Arabia in the period between January to September 2022. The GHRd3 polymorphism was genotyped using multiplex PCR. The correlation between height and genotypes was analyzed using one-way analysis of variance. The association between GHRd3 polymorphism and T2DM was assessed using logistic regression analysis.

**Results:**

The data showed a significant difference between the means of heights associated with each GHRd3 genotype, flfl, fld3, and d3d3. Logistic regression analysis showed no association between GHRd3 variants and T2DM.

**Conclusion:**

Homozygous GHRd3 polymorphism carriers, d3d3 genotype, were taller than fld3 or flfl carriers in our population. None of the GHRd3 variants were associated with T2DM. Thus, the GHRd3 polymorphism has growth-related actions with a minor contribution to T2DM. However, more studies with a larger sample size are required to confirm these findings.

## INTRODUCTION

Growth hormone (GH)-insulin-like growth factor-I (IGF-I) axis regulates human postnatal growth and glucose metabolism.^1^ Human GH signals via growth hormone receptor (GHR), a transmembrane protein dimer belonging to the type-I cytokine receptor superfamily.^2^ Full-length GHR is encoded by the human *GHR* gene located on the short arm of chromosome 5, with nine coding exons.^2^ Deletion of exon-3 from the GHR gene (GHRd3) is a common human-specific polymorphism, which results in the absence of 23 amino acids from the GHR extracellular domain.^3^ Deletion of exon-3 appears to influence membrane trafficking and stability of GHR; however, the GH-binding ability remains intact.^4^ The impact of the GHRd3 polymorphism on GHR signaling was first seen as an increased sensitivity to recombinant human GH (rhGH) therapy.^5^ The GHRd3 polymorphism has been shown to increase growth responses to human recombinant GH (hGH) treatment in children with or without GH deficiency (GHD).^6^ Patients with GHD who are homozygous for the GHRd3 polymorphism (d3/d3 genotype) are highly responsive to hGH therapy.^7^ In another study, children with idiopathic short stature carrying the homozygous GHRd3 (d3/d3) genotype exhibited increased sensitivity to hGH treatment presented by higher short-term IGF-1 production.^8^ The GHR d3 allele was also associated with faster postnatal growth in low-birth-weight preterm infants and small for gestational age children (SGA).^5^,^9^ However, regardless of the large number of studies reporting the effect of the GHRd3 polymorphism on human growth under some conditions, the direct effect of the GHRd3 polymorphism on the final adult height in the general population remains to be investigated. The sensitive GHRd3 allele may also be associated with increased male reproductive function such as high semen quality and sex-hormones levels 10 and a decreased risk of breast cancer.^11^

The GHRd3 polymorphism has also been implicated in the pathogenesis of insulin resistance and type-2 diabetes mellites (T2DM). For example, the GHRd3 allele was linked with increased insulin secretion and sensitivity during puberty.^12^ In acromegaly, this polymorphism was significantly associated with an increased body mass index (BMI) and insulin resistance.^13^,^14^ Additionally, in a study investigating the association between the GHRd3 polymorphism and Type-2 diabetes mellitus (T2DM), the homozygous d3d3 genotype was found to prevent disease development whereas the presence of the full-length (fl) GHR genotype (fl/fl) was associated with phenotypes indicative of metabolic disorders such as high body mass index (BMI), elevated C-reactive protein (CRP), impaired lipid levels, and impaired glucose tolerance.^15^

Diabetes mellitus (DM) is a multifactorial condition that is becoming a global health concern because of its rapidly increasing prevalence. According to the World Health Organization (WHO), approximately seven million individuals in Saudi Arabia are diabetic and more than three million are pre-diabetic, making Saudi Arabia the second-highest ranking country for diabetes prevalence in the Middle East.^16^ Type-2 diabetes mellitus (T2DM) is the most common form of diabetes characterized by hyperglycemia due to insulin resistance. The role of GH-IGF-1 in insulin resistance is well established. For instance, increased IGF-1 plasma levels are associated with reduced T2DM risk, and IGF-1 gene polymorphisms have been linked to T2DM and other age related disorders.^17^-^19^ Therefore, altered GHR responsiveness caused by GHR exon-3 deletion alleles may influence IGF-1 secretion levels and contribute to T2DM. Further, a shorter adult phenotype has been linked with an increased risk of T2DM.^20^ However, the relationship between the GHRd3 polymorphism and T2DM and adult height has not been investigated in Saudi Arabia. In this study, we investigated, for the first time, the genetic relationship of the GHRd3 polymorphism with adult height and T2DM in Jazan province, Saudi Arabia.

## METHODS

In total, 284 participants were included in this study and divided into two age-matched groups: i) 142 subjects previously diagnosed with T2DM and ii) 142 healthy control subjects. Patients were recruited from the outpatient clinic at Jazan University Hospital, Southwest Saudi Arabia, between January 2022 and September 2022. The inclusion criteria were as follows: Saudi Arabian citizens aged 35 years or older, with T2DM, or without T2DM or any other cardiovascular disease.

### Ethical approval

All participants signed a consent form before data or blood sample collection. Ethical approval was obtained from the local ethics approval committee (IRB No. REC-43/02/017) and the study was conducted according to the principles of the Declaration of Helsinki.

Basic data including age, sex, and a family history of T2DM were obtained through direct interviews. The volunteers’ weight in kilograms (kg) and height in centimeters (cm) was determined using the health o meter Professional scale 501KL (McCook, IL, USA). Height and weight measurements were obtained with bare feet and were repeated twice; the mean of the two measurements was used as the final height and weight. The body mass index (BMI) was calculated using weight (kg) and height (m) as follows: BMI = kg/m^2^. Fasting plasma glucose (FPG) levels were determined using the glucose oxidase method. Glycated hemoglobin (HbA1c) levels were determined using a quantitative turbidimetric inhibition immunoassay.

### DNA extraction and genotyping

Whole blood samples were collected from each patient in 5-mL EDTA tubes. Genomic DNA was extracted from 200 µL of whole blood using the GeneJET Genomic DNA Purification Kit (Thermo Scientific, Waltham, Massachusetts, USA) according to the manufacturer’s instructions. The DNA quantity was estimated using a NanoDrop microvolume spectrophotometer (Thermo Scientific). The ratio of absorbance at 260 and 280 nm (A_260_/A_280_) was determined to check the quality of the extracted DNA. Samples with ratios of 1.8 or higher were considered suitable for subsequent analysis. The full-length allele (fl) and exon 3-deleted allele (d3) of GHR were genotyped by multiplex polymerase chain reaction (PCR) as described previously.^21^

### Statistical analysis

Data are presented as mean ± standard deviation (SD) or percentage. Statistical comparisons between two groups were performed using the student’s t-test. Multiple group comparisons were performed using parametric one-way analysis of variance (ANOVA) with Tukey’s post-hoc test. To assess the relationship between the GHRd3 polymorphism and T2DM, binary logistic regression analysis was performed using the fl/fl genotype as a reference. Statistical significance was set at a *p*-value < 0.05. All statistical analyses were performed using GraphPad Prism-9 (GraphPad Software Inc., San Diego, CA, USA).

## RESULTS

The baseline characteristics of the control and T2DM groups are summarized in (Table-I). There was no significant difference in sex distribution between the two groups (*p* = 0.218). However, there was a significant difference in age (*p <* 0.0001), potentially because of the presence of a large proportion of older participants in the T2DM group. Upon comparing the heights of the two groups, a lower mean height (cm) was found in the T2DM group (161.2 ± 8.6) than in the control group (163.2 ± 6.8), which reached the statistical significance (*p* = 0.039). Patients with T2DM also demonstrated significantly higher mean BMI, fasting plasma glucose, and HbA1c levels than controls.

**Table-I:** Baseline characteristics of the study population.

Characteristic	Total n=284, 100%)	Control n= 142, 50%)	T2DM n= 142, 50%)	p-value
Gender n (M/F)	(132/152)	(62/80)	(70/72)	0.218*
Age (years)	54.1±14.3	49.2±13.9	59.0±13.1	<0.0001
Height (cm)	162.3±7.8	163.2±6.8	161.2±8.6	0.039
Weight (kg)	72.0±12.4	70.4±10.5	73.9±14.2	0.021
BMI (kg/m2)	27.3±4.4	26.5±4.0	28.4±4.7	<0.001
FPG (mmol/L)	8.8±4.9	5.6±1.0	12.1±5.3	<0.0001
HbA1c (%)	7.16±2.2	5.50±0.8	8.9±1.8	<0.0001

***Notes:*** Data are mean±SD unless otherwise specified. BMI; body mass index, FPG; fasting plasma glucose, HbA1c; Glycated hemoglobin.

* Chi-square test.

In the entire study population, the frequencies of GHRd3 variants were 44.4% (flfl), 43.0% (fld3), and 12.6% (d3d3) (Table-II). The Hardy-Weinberg equilibrium (HWE) test showed a *p*-value of 0.449, indicating that the genotype frequencies were at equilibrium. Similar distributions of GHRd3 variants were observed in the T2DM and control groups (Table-II). Additionally, logistic regression analysis based on genotype or allele analysis showed crude ORs closer to 1.0 and *p*-values > 0.05, indicating no significant association between the GHRd3 polymorphism and T2DM risk.

**Table-II:** Frequency of the GHRd3 polymorphism in the study groups and association with T2DM.

Genotype/Allele	Total n=284 n (%)	Control n = 142 n (%)	T2DM n = 142 n (%)	HWE p-value*	OR (95% CI) p-value**
flfl	126 (44.4)	65 (45.8)	61 (43.0)	0.449	Reference (1.0)
fld3	122 (43.0)	60 (42.2)	62 (43.7)		1.10 (0.68 -1.79) 0.799
d3d3	36 (12.6)	17 (12.0)	19 (13.4)		1.19 (0.58-2.50) 0.707
fld3+d3d3	158 (55.6)	77 (54.2)	81 (57.0)		1.12 (0.71-1.77) 0.720
fl	374 (65.8)	190 (66.9)	184 (64.8)		Reference (1.0)
d3	194 (34.2)	94 (33.1)	100 (35.2)		1.09 (0.78 - 1.55) 0.658

***Notes:*** T2DM: Type-2 Diabetes Mellitus. OR; Odds ratio. CI; confidence interval. HWE; Hardy-Weinberg equilibrium.

* Chi-square test (2-tailed).

** Fisher’s exact test (2-tailed).

**Fig.1:**
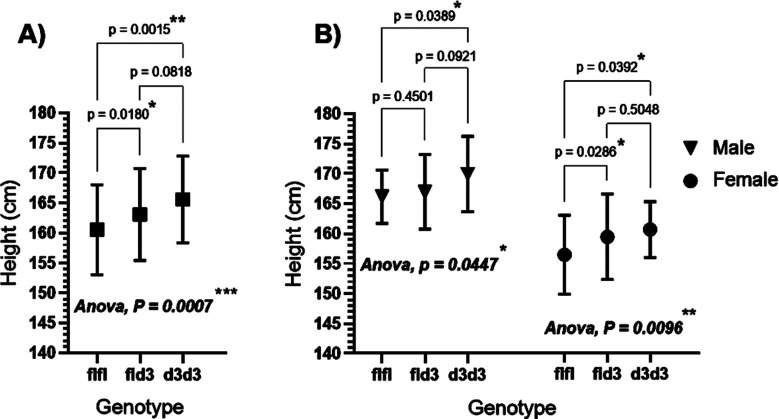
GHRd3 polymorphism and adult height in (A) total study population (***n***= 284) and (B) male (***n***=132) and female (*n*=152) populations separately. A: the heights (mean±SD) of individuals for each GHRd3 genotype was; flfl (160.5±7.50 cm), fld3 (163.1±7.68 cm), and d3d3 (165.6±7.21). B: in males, heights were; 166.2±4.46 cm (flfl), 167.0±6.24 cm (fld3), 169.9±6.28 cm (d3d3). In females, heights were; flfl;156.5±6.58 cm (flfl), 159.5±7.14 cm (fld3), and 160.7±4.65 cm (d3d3).

To determine the effect of the GHRd3 polymorphism on final adult height, the mean height of the total study population was plotted based on the GHRd3 genotypes (Fig.1A). One-way ANOVA showed a statistically significant difference between the means (*p*-value = 0.0007). The mean heights of individuals with the fld3 or d3d3 genotypes were 2.6 cm (*p* = 0.0180) or 5.1 cm (*p* = 0.0015) taller than those with the flfl genotype, respectively. To rule out the potential coincidence created by differences in sex distribution, data were further divided by sex, and the mean heights were re-plotted against each GHRd3 genotype; a similar effect was also observed (Fig.1B).

## Discussion

The distribution of GHRd3 polymorphisms in Saudi Arabia has been published elsewhere.^21^ In this study, we investigated the association between GHRd3 polymorphisms and adult height or T2DM in the Saudi Arabian population from the Jazan area. To our knowledge, this is the first study to explore this topic in this region. Our data initially showed a positive relationship between the GHRd3 polymorphism and final adult height. Carriers of the full-length GHR genotype (flfl) demonstrated shorter phenotypes than those of the fld3/d3d3 genotype, regardless of sex. This finding may be explained, at least partially, by the increased sensitivity of GHRd3 to human GH. This phenomenon has been confirmed in multiple studies on patients with GH deficiency, Turner’s syndrome, neonates born small for their gestational age, and children with idiopathic short stature.^5^,^8^,^22^,^23^ These studies have demonstrated increased responsiveness to recombinant human GH therapy in homozygous d3d3 genotype carriers compared to that in fld3 or flfl genotype carriers, as indicated by accelerated longitudinal bone growth and increased final height. However, in contrast some studies on patients with acromegaly have reported no effect of this polymorphism on adult height.^13^,^24^ Furthermore, a review-based study analyzed the effect of the GHRd3 polymorphism on weight, height, IGF-1 levels, and cardiovascular risk factors from 31 published articles in different populations and concluded a lack of association between the GHRd3 polymorphism and any of these parameters.^25^

Notably, we found a significant relationship between the shorter adult phenotype and T2DM in our population. The relationship between adult height and T2DM remains inconclusive and contradictory. This is further exacerbated by the fact that most studies on T2DM have focused on BMI rather than height. However, several studies have reported findings similar to our observations in this study. The first study, published in 1998, reported an association between short stature and T2DM in a population from the Netherlands.^26^ This observation was further supported by multiple studies on different populations in the following years.^27^-^29^ Furthermore, these reports were strengthened by a recently published meta-analysis, which concluded that shorter adult height is associated with an increased risk of T2DM.^20^

In this study, we did not observe any association between GHRd3 variants and the likelihood of developing T2DM. This finding contradicts some previously published reports and it should not eliminate the possibility of a potential role of GHRd3 in the pathogenesis of T2DM in some populations. For instance, a study conducted by Strawbridge et al. on a Swedish population concluded that GHR d3 allele carriers were less likely to develop T2DM.^15^ Additionally, the GHR d3 allele is associated with increased insulin sensitivity, reduced obesity, and other metabolic risk factors for T2DM in obese Chinese children.^30^

### Limitations

The relatively small sample size was a major limitation of this study. Additionally, owing to technical limitations, we could not assess the plasma levels of some biomarkers, such as IGF-1, to evaluate GHR activity in different GHRd3 genotypes. Therefore, additional studies with a larger sample size and additional blood or serum chemical biomarkers are required to confirm our findings.

## Conclusion

This study determined the frequency of several GHRd3 genotypes in the general population and in patients with T2DM in Saudi Arabia. We found for the first time that the GHRd3 polymorphism may contribute directly and independently to the final adult height in our population from the Jazan region. However, this study showed no evidence of a direct relationship between the GHRd3 polymorphism and T2DM development. Therefore, this polymorphism may not be a risk factor for T2DM development.

### Authors Contribution:

**YAK:** Designed and did statistical analysis, editing and reviewing of the manuscript, and he is responsible for integrity of research.

**RGH**, **AYM** & **RAAS:** Did blood samples and data collection and drafted the manuscript.
